# Bcl6 Sets a Threshold for Antiviral Signaling by Restraining IRF7 Transcriptional Program

**DOI:** 10.1038/srep18778

**Published:** 2016-01-05

**Authors:** Feng Xu, Yanhua Kang, Ningtong Zhuang, Zhe Lu, Hang Zhang, Dakang Xu, Yina Ding, Hongping Yin, Liyun Shi

**Affiliations:** 1 Department of Infectious Diseases, Second Affiliated Hospital, Zhejiang University School of Medicine, Hangzhou, Zhejiang 310009, China; 2 Department of Microbiology and Immunology, Key Lab of Immunology and Molecular Medicine, School of Medicine, Hangzhou Normal University, Hangzhou, Zhejiang 310036, China; 3 MIMR-PHI Institute of Medical Research, Clayton, Victoria 3168, Australia; 4 Department of Microbiology and Immunology, Nanjing University of Chinese Medicine, Nanjing 210046, China

## Abstract

The coordination of restraining and priming of antiviral signaling constitute a fundamental aspect of immunological functions. However, we currently know little about the molecular events that can translate the pathogenic cues into the appropriate code for antiviral defense. Our present study reports a specific role of B cell lymphoma (Bcl)6 as a checkpoint in the initiation of the host response to cytosolic RNA viruses. Remarkably, Bcl6 specifically binds to the interferon-regulatory factor (IRF)7 loci and restrains its transcription, thereby functioning as a negative regulator for interferon (IFN)-β production and antiviral responses. The signal-controlled turnover of the Bcl6, most likely mediated by microRNA-127, coordinates the antiviral response and inflammatory sequelae. Accordingly, de-repression of Bcl6 resulted in a phenotypic conversion of macrophages into highly potent IFN-producing cells and rendered mice more resistant to pathogenic RNA virus infection. The failure to remove the Bcl6 regulator, however, impedes the antiviral signaling and exaggerates viral pneumonia in mice. We thus reveal a novel key molecular checkpoint to orchestrate antiviral innate immunity.

Antiviral immunity is presumed to be initiated by pattern recognition receptors (PRRs) on host cells to specifically sense pathogen-associated molecular patterns (PAMPs) of invading viruses. This recognition subsequently leads to the mobilization of signaling cascades and ultimately the generation of type I interferon (IFN) and interferon-stimulated genes (ISGs)^1^,^2^. Among the known PRRs, retinoic acid-inducible gene-I (RIG-I), a member of the RIG-I-like receptors (RLRs), functions to recognize viral short dsRNA and 5′-triphosphate panhandle RNA in the cytoplasm^3^. Upon binding to viral RNA, RIG-I undergoes a conformational change and interplays with the mitochondrial adaptor protein, virus-induced signaling adapter (VISA), which in turn recruits TANK-binding kinase 1 (TBK1)/inducible I κappa-B kinase (IKKi) and phosphorylates IFN regulatory factor (IRF)3 and IRF7. Phosphorylated IRF3 and IRF7 then dimerize and translocate into the nucleus to trigger type I IFN production^4^. Additionally, VISA associates with TNF receptor associated factor 6 (TRAF6) and activates NF-kappaB (NF-κB) through the kinase IKKα/β^5^. Thus, activated IRF3/7 and NF-κB collaboratively induce the activation of innate immune genes upon RIG-I engagement^3^,^6^.

IRF3 and IRF7 both are master transcription factors required for antiviral immunity. Compared with IRF3, which is constitutively expressed in the cytosol, IRF7 is likely induced at the transcriptional level following viral exposure. The central role of IRF7 in the immune system has been highlighted by the pathology observed in the IRF7 deficient mice, which displayed a strikingly blunted type I IFN response and extensive viral infection^7^. In contrast, deletion of the inhibitors of IRF7 synthesis, eukaryotic translation initiation factor 4E-binding protein (EIF4EBP) 1 and EIF4EBP2, resulted in the restorative expression of IFN and the protection against severe viral diseases^8^. Due to its significance in delivering antiviral signaling, IRF7 is vulnerable to multiple layers of regulatory machinery, such as ubiquitination, sumoylation and dimerization blocking^9^,^10^,^11^. Despite the advance in our understanding of virus detection and signaling transduction, the knowledge about the regulation of antiviral signaling, particularly at the IRF7 level, is still quite limited.

B cell lymphocyte 6 (Bcl6) is a member of the BTB (BR-C, ttk and bab)/POZ (Pox virus and Zinc finger) family and a master transcriptional regulator essential for various physiopathologies^12^. It was originally characterized as an oncogene in diffuse large B cell lymphoma (DLBCL) and was also found in some solid tumors, such as bladder, colon cancer and hepatoma^13^. Accumulating evidences showed that Bcl6 played a pivotal role in lymphocyte development and immune responses, and the Bcl6 null mice failed to form germinal centers or mount T-cell-dependent antibody responses^14^. Additionally, Bcl6 was found to potentially inhibit the production of proinflammatory cytokines and chemokines upon toll-like receptor (TLR) engagement, presumably due to its ability to antagonize NF-κB activity^15^. Intriguingly, the aberrant expression of Bcl6 has been implicated in some of virus-associated diseases such as Epstein-Barr virus (EBV)-positive Burkitt lymphoma and hepatitis C virus (HCV)-related hepatocellular carcinoma (HCC), suggesting a potential role for Bcl6 in viral control^16^,^17^,^18^. However, in contrast to its well-established role in carcinogenesis and lymphocyte development, the function of Bcl6 in antiviral responses has never been investigated.

MicroRNA (miRNA) is a class of endogenously expressed small noncoding RNAs, which serves to regulate the gene expression by modulating both the stability and translation of mRNAs^19^. Although the action mode of miRNAs is reasonably well known, studies on the interplay between miRNAs and viruses have just begun to unravel. The expression of certain cellular miRNAs could be advantageous or disadvantages to viral infection, through their interaction with viral mRNAs or the antiviral signaling/effector molecules^20^,^21^. MiR-127 was previously found to be highly expressed in embryos and essential for lung development, placental formation and cellular apoptosis^22^. Our previous study indicated that miR-127 can specifically target Bcl6 expression and promote the development of inflammatory macrophages, suggesting its potential role in immune regulation^23^. Recently, evidences are emerging to implicate miR-127 in viral pathology. For example, miR-127 has been induced by Epstein-Barr Virus (EBV) and played a role in regulating B cell development and thus antiviral immunity^24^. Also, HBV infection can elevate the expression of miR-127, which then functioned to fine–tune HBV behavior via modulating endoplasmic reticulum (ER) stress^25^. Despite these findings, the exact role of miR-127 in antiviral process and the underlying mechanism are largely undefined.

In this study, we demonstrate a prominent role for Bcl6 in the initiation of RIG-I-driven antiviral immunity, particularly under control of miR-127. Remarkably, we find that Bcl6 functionally interacts with nuclear receptor co-repressor (NcoR)2 and histone deacetylase (HDAC)3, to direct repressive histone marks on IRF7 and set a check on antiviral signaling. Active de-repression of the antiviral program is largely dependent on the induction of miR-127, which, upon viral infection, potentially suppresses the expression of Bcl6 and impairs the inhibitory complex at IRF7 loci. Thus, we, for the first time, establish a very specific role of the Bcl6 in antiviral response and provide a novel mechanism whereby a proto-oncogenic protein instructs host antiviral defense.

## Results

### Bcl6 is a negative regulator of RIG-I-mediated antiviral response

**Figure 1:**
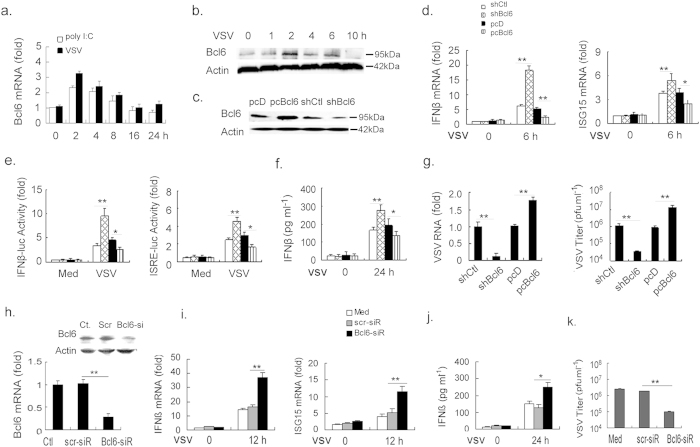
Bcl6 negatively regulates type I IFN production upon RNA virus infection. (**a,b**) Murine peritoneal macrophages from C57BL/6 were transfected with poly I:C (1 μg ml^−1^) or infected with VSV (MOI 1) for the indicated hours. mRNA and protein levels of Bcl6 were detected by quantitative PCR (qPCR) or immunoblot respectively. qPCR results were presented as folds to the endogenous control β-actin. **(c)** RAW264.7 cells were stably transfected with control small hairpin RNA (shCtl) or shBcl6, pcDNA (pcD) or pcDNA-Bcl6 (pcBcl6) expression vectors. The efficiency of knockdown and overexpression of Bcl6 was examined by immunoblot. **(d–g)** RAW264.7 cells stably transfected with shCtl or shBcl6, pcD or pcBcl6 were infected with VSV (MOI 1) for 6 h. mRNA level of IFNβ and ISG15 was examined by qPCR **(d)**; The activity of IFNβ and ISRE promoters was tested by dual-luciferase assay 6 h post infection and the value was normalized to renilla luciferase activity **(e)**; The protein level of IFNβ in cell suspension was analyzed by ELISA **(f)**, and viral titers and RNA level 24 h post infection were examined as described in material and methods **(g)**. (**h**) THP-1 cells were transfected with Bcl6-specific or scrambled siRNA for 48 h, and Knockdown efficiency was analyzed by qPCR or immunoblot. (**i–k**) The cells in **(h)** were challenged with VSV (MOI, 1) for 24 h. qPCR analysis of IFNβ and ISG15 mRNA (i) and ELISA assay of IFNβ protein **(j)** were performed, and viral titers were assessed by standard plaque assays **(k)**. Data are representative of three experiments and depicted as means ± SEM. **p < 0.01, *p < 0.05 by student’s *t* test. Results obtained from immunoblot are representative of three independent experiments. Uncropped images are shown in Supplementary Information.

To define the potential role of Bcl6 in host defense against RNA viruses, we first analyzed its expression in murine macrophages in response to viral triggers. The result showed that, upon vesicular stomatitis virus (VSV) infection or intracellular poly I:C stimulation, Bcl6 was rapidly induced at the very early stage of challenge, followed by a reduction at later time period (Fig. 1a,b). This expression profile appeared to be inversely correlated with that of IFNβ, suggesting the possible involvement of Bcl6 in antiviral regulation. We thus set out to study the effect of Bcl6 on antiviral response driven by RIG-I, a key innate sensor for RNA viruses. To this end, murine macrophage RAW264.7 cell lines were established to stably express or silence *Bcl6* gene (Fig. 1c). Further study indicated that, enforced Bcl6 expression profoundly decreased the expression of IFNβ and ISG15, the representative interferon-stimulated gene, upon VSV infection. Conversely, Bcl6 knockdown in macrophages caused a remarkable up-regulation of IFNβ and ISG15 (Fig. 1d). Consistently, the activity of IFNβ- or interferon-stimulated response element (ISRE)-driven promoter was inhibited by Bcl6 overexpression but enhanced upon Bcl6 silencing following VSV stimulation (Fig. 1e). Also, the secretion of IFNβ was significantly decreased in Bcl6-deficient cells while increased in Bcl6-expressing cells, inversely correlating with the viral load detected in these cells (Fig. 1f,g). Moreover, we tested the effect of Bcl6 on the response of human monocyte THP-1 cells to VSV infection. We firstly knocked down *Bcl6* gene in THP-1 cells by RNA-mediated interference and the efficiency was confirmed (Fig. 1h). The cells were then subjected to VSV infection. Expectedly, the expression of IFNβ and ISG15 was remarkably enhanced upon Bcl6 silencing in THP-1 cells and the viral burden was accordingly decreased (Fig. 1i–k). Together, these results indicate a dominant role for Bcl6 in the regulation of RIG-I-triggered antiviral response both in human and murine innate immune cells.

### Bcl6 targets IRF7 to repress antiviral signaling

**Figure 2:**
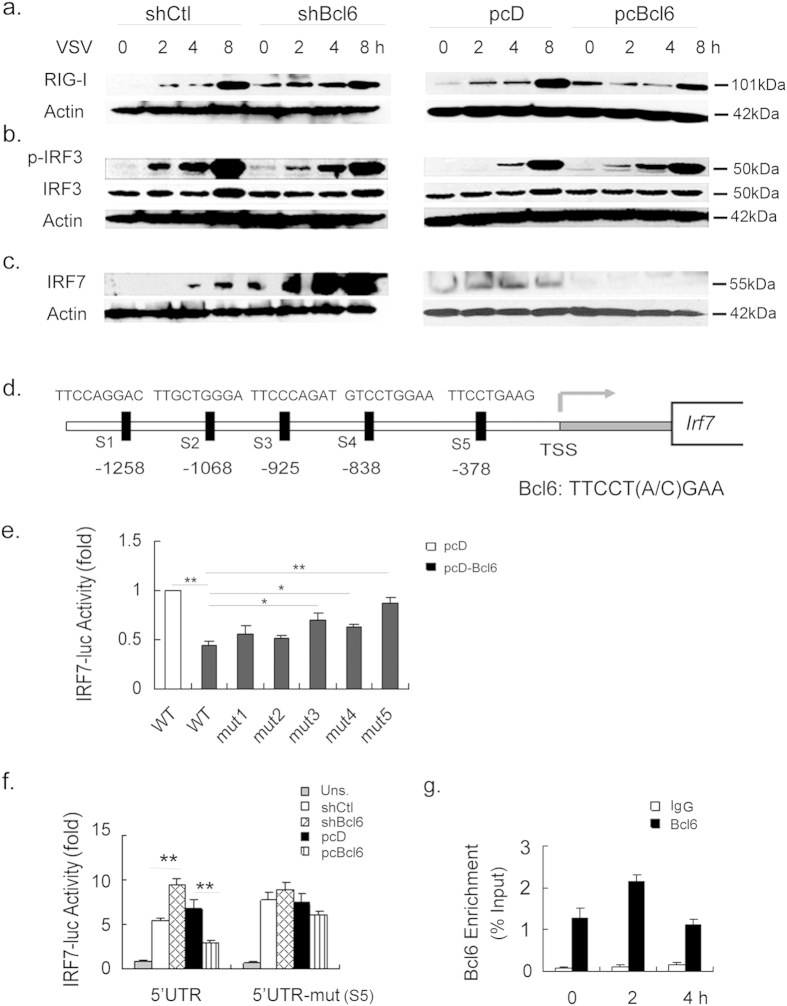
Bcl6 specifically modulates IRF7-driven antiviral signaling. RAW264.7 cells transfected with shCtl or shBcl6, pcD or pcBcl6 were infected with VSV (MOI 1) for the time period indicated. (**a–c**) Immunoblot analysis of the total or phosphorylated (p-) proteins in cell lysates. (**d**) Sequences (S1–S5) of the potential Bcl6-binding sites were given in comparison with the 9-bp consensus Bcl6-binding site. TSS, transcription start site. (**e**) The promoter construct containing wide-type (WT) IRF7 or the constructs with the deletion of the predicted site respectively (mut1–5) were transfected into HEK293T cells, along with pcDNA-Bcl6 or pcDNA plasmids. Promoter activities were determined by dual-luciferase assay and the values were normalized to control cells that were co-transfected with pcDNA and WT promoter plasmid (blank column). (**f**) Analysis of IRF7 promoter activity by dual-luciferase assay in the indicated RAW264.7 cell lines that were transfected with the reporter constructs containing the intact or mutant (S5: −386 to −378) fragments of IRF7 5′UTR. The value was normalized to renilla luciferase activity. (**g**) CHIP analysis of binding of Bcl6 to the promoter of IRF7 in RAW 264.7 cells upon VSV infection. IgG was used as a negative control. The enrichment was calculated relative to the input. Data are representative of three experiments and depicted as means ± SEM. **p < 0.01, *p < 0.05 by student’s t test or two-way ANOVA analysis. Results obtained from immunoblot are representative of three independent experiments. Uncropped images are shown in Supplementary Information.

Next, we sought to identify the mechanism by which Bcl6 mediated RIG-I signaling regulation. It appeared that the Bcl6 manipulation in macrophages had little effects on the expression of RIG-I itself as revealed in this study (Fig. 2a). We then focused on IRF3 and IRF7, the two major transcriptional factors that are required for antiviral signaling and IFNβ production^3^. The result showed that Bcl6 exerted little effect on the activity and the expression of IRF3 (Fig. 2b). In contrast, IRF7 level was markedly repressed by Bcl6 overexpression and boosted by Bcl6 deletion in macrophages during viral infection (Fig. 2c), suggesting that selective regulation of IRF7 expression may contribute to Bcl6-mediated RIG-I signaling modulation. Since Bcl6 has been recognized as a master transcriptional factor for the key genes involved in biological activities, we speculated that Bcl6 may modulate IRF7 gene expression at the transcriptional level. In support of this hypothesis, we identified 5 DNA sequences in a ~1.6 kb IRF7 promoter region that resembled the canonical Bcl6 binding motifs (Fig. 2d). We then constructed the intact IRF7 promoter plasmid as well as the plasmids with mutation at the predicted binding site (S1–S5) respectively, and the promoter activity was subsequently tested in HEK293T cells. Strikingly, it was shown that IRF7-driven promoter activity was markedly suppressed by Bcl6 overexpression (Fig. 2e). The mutation of S1–S5 at 5′UTR of IRF7 exhibited distinct effect on the promoter activity, with the most profound effect at S5 (−386~−378). We then chose site5 to have further analysis. Using the established RAW264.7 cell lines, we further confirmed that IRF7 promoter activity was repressed in Bcl6-expressing macrophages while increased in Bcl6-silenced cells. However, this effect was largely abrogated by S5 mutation at IRF7 promoter (Fig. 2f). Moreover, with a CHIP assay, we demonstrated a marked Bcl6 binding to the IRF7 promoter, particularly during the initial stage of VSV infection (Fig. 2g). The data thus confirmed that Bcl6 exerted a regulatory role on IRF7 expression in a direct DNA-binding manner.

### Bcl6 cooperates with NcoR and HDAC3 to restrain the early activation of IRF7

**Figure 3:**
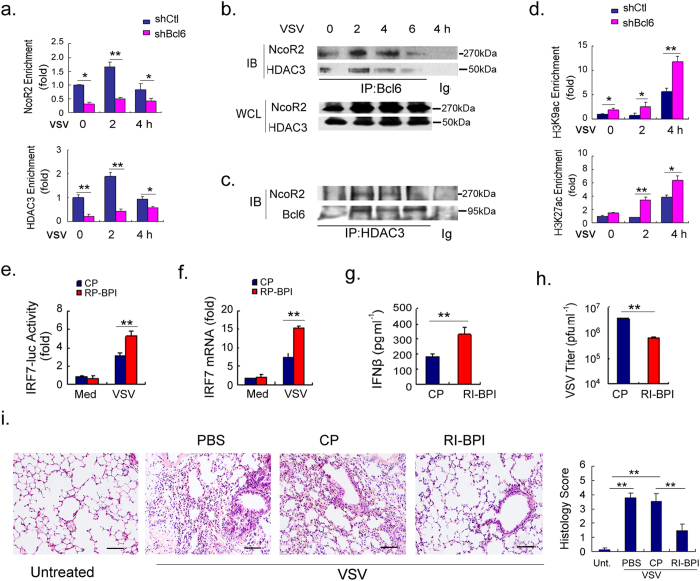
Bcl6 controls IRF7 gene transcription via interaction with HDAC3 and NcoR2. (**a**) CHIP analysis of binding of NcoR2 or HDAC3 to the promoter of IRF7 upon viral infection in RAW264.7 cells with or without Bcl6 knockdown. The enrichment of the indicted factor relative to Ig control was calculated as described in the Methods and the value was further normalized to control cells (shCtl) at the start of infection (0 h). (**b,c**) Co-immunoprecipitation analysis of the interaction of Bcl6 with NcoR2 and HDAC3 upon VSV infection. Immunoprecipitation (IP) with IgG was used as a negative control. Protein levels in whole cell lysates (WCL) were determined by immunoblot (IB). (**d**) ChIP analysis of histone modifications (H3K9ac, H3K27ac) at IRF7 promoter in RAW264.7 cells with or without Bcl6 knockdown. The enrichment of the indicted factor relative to Ig control was calculated as described in the Methods and the value was further normalized to control cells (shCtl) at the start of infection (0 h). (**e,f**) RAW264.7 cells were pretreated with 5 μM of control peptide (CP) or RI-BPI for 12 h, followed by VSV infection (MOI 1). The promoter activity and mRNA level of IRF7 were analyzed respectively 6 h post infections. (**g–i**) C57 BL/6 mice (n = 5 each group) were pretreated with RI-BPI or control peptide (10 mg kg^−1^, i.t.) for 6 h and then infected with VSV (2 × 10^7 ^pfu per mouse) for 24 h. IFNβ level in BALF was measured by ELISA **(g)**, the viral burden in lungs was determined by standard plaque assays **(**h**)**, and the representative H&E staining of lung tissues, as well as the histological scores were shown (**i**). Scale bar, 50 μm. The results are representative of three experiments (**a–f**). Data are depicted as means ± SEM. **p < 0.01, *p < 0.05 by student’s *t* test or two-way ANOVA analysis. Uncropped images are shown in Supplementary Information.

It is known that, in contrast to the constitutive IRF3 expression, IRF7 is presumed to be transcriptionally induced following viral exposure, and thus acts as a rate-limiting factor for IFN production^7^,^8^. Notably, we found that Bcl6 was specifically tethered at IRF7 loci in early stimulated or even unchallenged macrophages, and the level of Bcl6 likely determined its binding to IRF7 over the infectious period (Fig. 2f). Additionally, Bcl6 is known to associate with co-factors such as NcoR, BcoR and HDAC3, which are generally thought to be the critical components of the transcriptional regulatory complex^26^,^27^. We thus hypothesized that Bcl6 may cooperate with partner molecules to regulate the IRF7 transcriptional program and the subsequent antiviral response. To test it, we first used primers located at Bcl6 binding sites within IRF7 loci to examine the co-localization between Bcl6 and NcoR2, HDAC3. The result showed that, in parallel to that of Bcl6, dynamic binding of NcoR2 and HDAC3 to IRF7 promoter occurred in the early infectious phase. RNAi-mediated interference of Bcl6, however, abolished the NcoR2 and HDAC3 tethering to the IRF7 promoter, suggesting that the cofactor binding was Bcl6-dependent (Fig. 3a). The direct interaction between Bcl6 and NcoR2 or HDAC3 was further confirmed in VSV-triggered macrophages. Notably, this conjugation appeared to be reduced with Bcl6 down-regulation over the infectious period but irrelevant to the abundance of NcoR2 and HDAC3 which remained high level throughout (Fig. 3b,c). Thus, the data for the first time revealed the capability of Bcl6 to recruit partner molecules and form a regulatory complex in controlling IRF7 gene transcription.

Since the histone deacetylase, HDAC3, can be able to modify the chromatin state by altering local histone acetylation and thus regulate the target gene transcription^28^,^29^, we next analyzed the chromatin state of IRF7 gene upon Bcl6 regulation. The results showed that the acetylation of IRF7 at H3K9 and H3K27, the indicative of gene activation, was suppressed in WT macrophages as compared with that in Bcl6-silenced cells (Fig. 3d). This appeared to be inversely correlated with the binding of HDAC3 at IRF7 promoter in these two groups of cells (Fig. 3a). Clearance of the repressive complex by Bcl6 silencing, however, caused a significant elevation in H3K9 and H3K27 acetylation (Fig. 3d). The data thus suggested that HDAC-mediated modulation of chromatin accessibility may account for Bcl6-mediated regulation of antiviral gene. In support of this, pharmacological interference with the HDAC inhibitors valproic acid (VPA) or apicidin resulted in de-repression of IRF7 transcription in macrophages (supplementary Fig. 1a,b). More precisely, HDAC3 depletion caused a dramatic increase in IRF7 promoter activity (supplementary Fig. 1c), further confirming the inhibition of IRF7 transcription by HDAC3.

Next, to further confirm that it was the Bcl6 co-regulator complex rather than Bcl6 alone that mediated this regulation, we used RI-BPI^30^, a peptide developed to specifically block the interaction between Bcl6 and NcoR2 in the study. Remarkably, RI-BPI treatment caused a profound increase in IRF7 transcription in murine macrophages in response to VSV infection, whereas a similar cell penetrating control peptide (CP), which lacked the Bcl6 binding domain, had no apparent effect (Fig. 3e,f). More strikingly, in the VSV pulmonary infection model, RI-BPI treatment significantly enhanced IFNβ production and thus reduced the virus loads in mouse lungs compared with the control peptide treatment (Fig. 3g,h). This was associated with the improved symptoms of viral pneumonia (Fig. 3i), indicating that disruption of the interaction between Bcl6 and co-factors de-repressed the antiviral signaling and conveyed the protection against viral infection *in vivo*.

Taken together, our finding revealed that, by recruitment of the nuclear receptor NcoR2 and the chromatin modifier HDAC3, Bcl6 formed a co-repressive complex at the IRF7 promoter. Signal-dependent clearance of this co-repressor was essential for remodeling of chromatin state and activating the gene transcription program that was required for host antiviral activity.

### Bcl6 promotes the antiviral inflammatory sequelae

**Figure 4:**
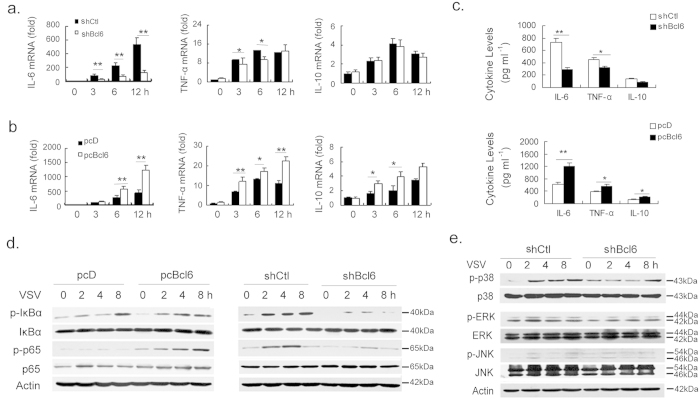
Enhancement of the inflammatory sequelae of antiviral responses by Bcl6. RAW264.7 cells stably transfected with pcBcl6 or pcD, shBcl6 or control shRNA were infected with VSV (MOI 1) for the time periods as indicated. (**a,b**) mRNA levels of IL-6, TNF-α and IL-10 were analyzed by qPCR. Results were presented as folds to the endogenous control β-actin. (**c**) Protein levels of IL-6, TNF-α and IL-10 in the supernatants were examined by ELISA 12 h post infection. (**d,e**) Phosphorylated (p-) or total p65, IκBα and MAPKs were detected by Immunoblot analysis at the indicated time periods. Results are representative of three experiments. The data are depicted as means ± SEM. **p < 0.01, *p < 0.05 by student’s t-test (**a–c**). Uncropped images are shown in Supplementary Information.

The optimal antiviral defense requires the appropriate balance between immune response and lateral inflammatory damage. We next evaluated the role of Bcl6 in VSV-triggered inflammatory signaling. Unexpectedly, we found that Bcl6 overexpression significantly increased the expression of the pro-inflammatory cytokines such as IL-6 and TNF-α, whereas loss of Bcl6 inhibited their transcription. Similarly, the immune-modulatory cytokine IL-10 was also increased by Bcl6 overexpression and decreased by Bcl6 loss, though to a lesser degree (Fig. 4a–c). These data may suggest a positive role for Bcl6 in NF-κB-driven signaling regulation. Indeed, it was shown that NF-κB and IκBα activation, the key signaling events for inflammatory signaling, were remarkably enhanced by Bcl6 overexpression and suppressed by Bcl6 deficiency upon VSV challenge (Fig. 4d). However, little effect of Bcl6 was observed on VSV-triggered activation of mitogen-activated protein kinases (MAPKs), including ERK, JNK and p38 kinase (Fig. 4e). Notably, this proinflammatory property of Bcl6 appeared to be congruent with the observed effect of the Bcl6 blocking peptide, RI-BPI, which potentially alleviated the inflammatory pathology in mouse model of viral pneumonia (Fig. 3i). Together, we proposed that Bcl6 played a previously unappreciated but critical role in promoting the inflammatory sequelae of antiviral responses.

### MiR-127 mediates signal-dependent turnover of the Bcl6 co-regulator

**Figure 5:**
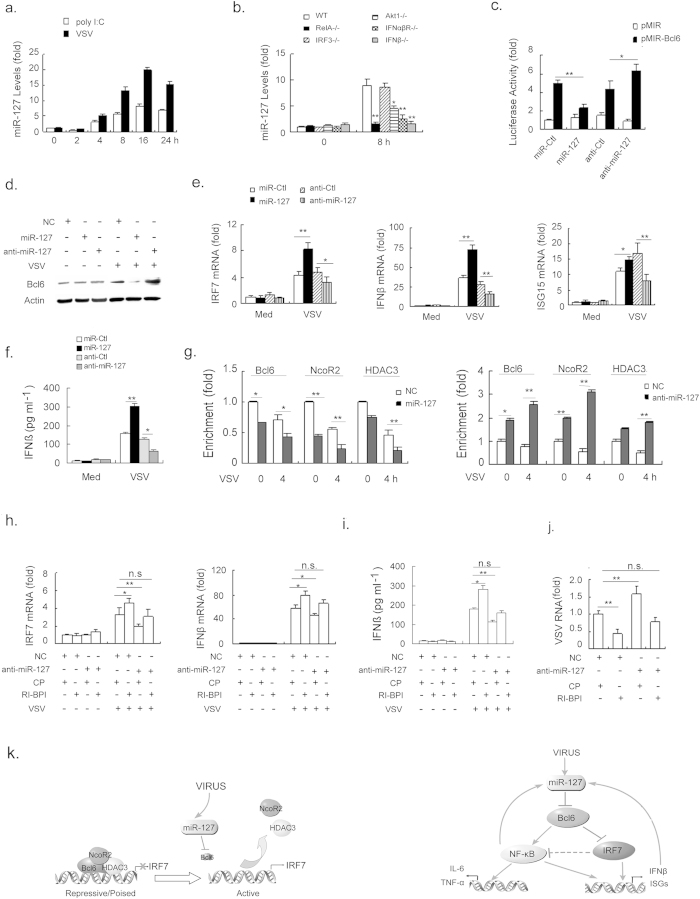
miR-127 mediates the signal-dependent turnover of Bcl6 coregulator. (**a**) mRNA level of miR-127 was examined in primary peritoneal macrophages transfected with poly I:C (1 μg ml^−1^) or infected with VSV (MOI 1) for the indicated hours. U6 was used as an endogenous control. (**b**) The induction of miR-127 upon VSV infection was examined in mouse peritoneal macrophages deficient in NF-κB, IRF3, Akt1, IFNβ or IFNαβR. (**c**) Luciferase activity in RAW264.7 cells transfected with the reporter constructs containing 3′UTR of Bcl6, along with miR-127, anti-miR-127 or their controls respectively. The value was normalized to renilla luciferase activity. (**d–f**) RAW264.7 cells were transfected with miR-127, anti-miR-127 or their controls for 24 h and then infected with VSV for 8 h. Western blot analysis of Bcl6 protein level (**d**); qPCR analysis for mRNA levels of IRF7, IFNβ and ISG15 (**e**); and ELISA assays for IFNβ secretion (**f**); (**g**) The recruitment of Bcl6 (Bcl), NcoR2 (Nco) and HDAC3 (HD) onto IRF7 promoter were examined 8 h post infection. The enrichment of the indicted factor relative to Ig control was calculated as described in the Methods and the value was further normalized to control cells (NC) at the start of infection (0 h). (**h–j**) RAW264.7 cells were transfected with anti-miR-127 or NC, followed by 12 h treatment of RI-BPI or the control peptide (10 μM each). Cells were then infected with VSV for 8 h. qPCR analysis for mRNA level of IRF7 and IFNβ (**h**), ELISA assays for IFNβ (**i**) and quantitation of VSV RNA by qPCR (**j**). Data are representative of three experiments and depicted as means ± SEM. **p < 0.01, *p < 0.05 by student’s *t* test or two-way ANOVA analysis. (**k**) Proposed working model of Bcl6-mediated coordination of antiviral response.

Based on the above observation, if the Bcl6, NcoR2 and HDAC3 ternary complex kept the basal IRF7 transcription in check, how then does VSV stimulation overcome this inhibition to promote antiviral signaling? Interestingly, we reported in a previous study that VSV can trigger a prominent induction of miR-127, a miRNA molecule that has been implicated in Bcl6-mediated cancer development and other pathophysiology^23^,^31^. In fact, the current study demonstrated that, following a transient repression, miR-127 was rapidly induced upon stimulation of intracellular poly I:C or VSV (Fig. 5a). The expression of miR-127 likely paralleled to IFNβ production while correlating inversely with Bcl6 expression (Fig. 1a,b). Also, we found that VSV-induced miR-127 expression was severely impaired upon the loss of NF-κB or IFN, the two key factors for antiviral signaling amplification (Fig. 5b and supplementary Fig. 2), implying the involvement of the miR-127/Bcl6 axis in RIG-I signaling. Because the potential sequence bound by miR-127 has been previously identified at the 3′UTR of the Bcl6 gene^23^, we then tested its direct effect on Bcl6 using a reporter plasmid containing the predicted sequence. The results indicated that the activity of reporter plasmid containing the intact Bcl6 3′UTR was significantly repressed by miR-127 mimic but enhanced by its antimir (Fig. 5c). Consistently, VSV-induced Bcl6 expression was remarkably down-regulated by miR-127 overexpression but elevated by miR-127 inhibition (Fig. 5d). The data thus implied an involvement of miR-127/Bcl6 axis in antiviral responses. In support of this, ectopic miR-127 expression showed to significantly enhance VSV-triggered IRF7 transcription, as well as the expression of IFNβ and ISG15. In contrast, inhibition of miR-127 expression kept these antiviral genes in check (Fig. 5e,f). Interestingly, we noted that treatment of macrophages with miR-127 mimic or antimir led to a marked decrease or increase respectively in the transcription of pro-inflammatory cytokines (supplementary Fig. 3a,b), consistent with the regulatory role for Bcl6 in inflammatory signaling we described above.

Next, given the above finding that the Bcl6 co-repressor played a pivotal role in IRF7-driven antiviral signaling, we then wondered if miR-127 would participate in this regulatory mode in the course of viral infection. Indeed, the induction of miR-127 upon VSV infection, parallel to its inhibition of Bcl6 expression, led to a disengagement of the co-repressors, NcoR2 and HDAC3 from the IRF7 gene promoter (Fig. 5g). Enforced expression of miR-127 however further promoted the clearance of the Bcl6 co-repressors, whereas miR-127 inhibitors augmented the binding of Bcl6, NcoR2 and HDAC3 to the IRF7 promoter site. These data thus suggested that miR-127 was essential for the Bcl6-mediated transcription regulation and chromatin remodeling that was essential for antiviral response. More strikingly, when we used RI-BPI to block the interplay between Bcl6 and NcoR2, the enhanced restraint on IRF7 transcription caused by miR-127 antimir was largely abrogated, as evidenced by the restoration of IRF7 and IFNβ expression, and accordingly, the decreased viral burden (Fig. 5h–j). The data further confirmed that miR-127 exerted its regulatory effect by specifically acting on Bcl6 and the associated co-inhibitory complex.

Taken together, the miR-127/Bcl6 axis constituted dynamic regulatory machinery during antiviral response. That is, constitutive and initial induction of Bcl6 in the innate immune cells formed a repressive chromatin structure at the key antiviral signaling molecules, and thus established a threshold for antiviral signaling activation. A signal-dependent induction of miR-127, mostly through down-regulation of Bcl6, led to the disassembly of the co-repressive complex and thereby facilitated the signaling activation (Fig. 5k).

### The *in vivo* function of the miR-127/Bcl6/IRF7 regulatory circuit

**Figure 6:**
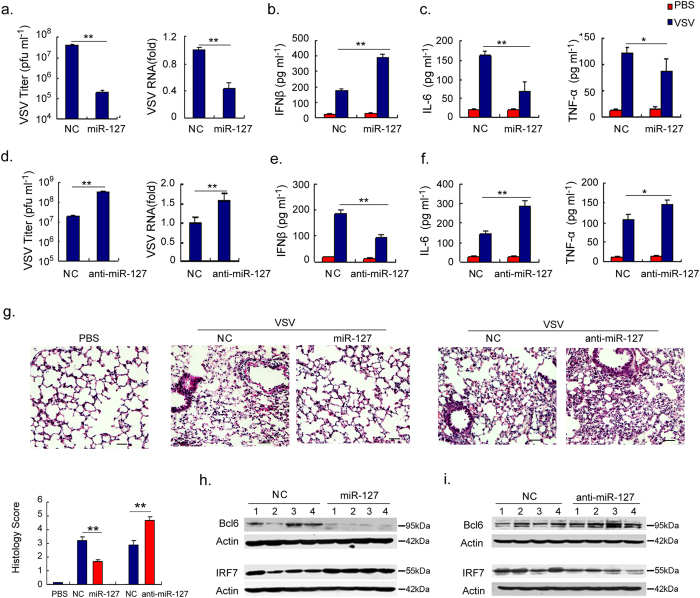
The miR-127-Bcl6-IRF7 circuit regulates the viral immunopathologic response *in vivo*. C57BL/6 mice (n = 4 per group) were pretreated with miR-127, anti-miR-127 or their non-specific controls for 24 h and then infected with VSV (2 × 10^7^ pfu per mouse). The animals were sacrificed 24 h later and the samples were collected for the functional analysis. (**a,d**) The virus titer in BALF or VSV RNA in lung tissues was examined respectively. (**b,e**) The levels of IFNβ, and (**c,f**) the levels of IL-6 and TNF-α in BALF were determined by ELISA. (**g**) Representative H&E staining of lung tissue sections and histological scores were shown (lower left). Scale bar, 20 μm. (**h,i**) Immunoblot analysis of Bcl6 and IRF7 levels in murine lung tissues; Data are depicted as means ± SEM. **p < 0.01, *p < 0.05 by student’s *t* test. Uncropped images are shown in Supplementary Information.

To further understand the functional significance of the miR-127/Bcl6/IRF7 circuit in host antiviral immunity, we investigated its role in a VSV pulmonary infection model, a typical antiviral immunity intrinsic to alveolar macrophages. Strikingly, pretreatment of mice with a miR-127 mimic, but not a scrambled control, significantly reduced viral loads in their lungs, in terms of viral titer and viral mRNA levels. In contrast, anti-miR-127 administration caused a profound elevation in the viral burden (Fig. 6a,d). Consistently, IFNβ level in BALF was raised by miR-127 treatment but repressed upon miR-127 inhibition (Fig. 6b,e). The expression of proinflammatory cytokines IL-6 and TNF-α, however, was suppressed upon miR-127 overexpression and increased by miR-127 blocking (Fig. 6c,f). Congruent with this, a substantial decrease in inflammatory cell infiltration and debris deposition was observed in miR-127-treated mice, whereas exaggerated inflammatory pathology was generated in the animals receiving miR-127 antimir (Fig. 6g). Importantly, we demonstrated that the protection conveyed by miR-127 was closely related with its ability to repress Bcl6 expression and thus enhance IRF7 gene (Fig. 6h,i). Together, our data demonstrated a prominent and specific role of the miR-127/Bcl6/IRF7 loop in the host innate immunity against viral infection.

## Discussion

In this study, we report a prominent and specific role for Bcl6 in the transcriptional modulation of RIG-I-driven antiviral response. We show that, in resting or transiently activated macrophages, Bcl6 cooperated with the co-factors NcoR2 and HDAC3 to occupy and inhibit the chromatin accessibility of IRF7 gene, thereby restraining innate antiviral signaling. By contrast, signal-dependent removal of the Bcl6 co-repressor relieved the IRF7 promoter from the transcriptional repression and facilitated the antiviral signaling and IFNβ production (Fig. 5k). Furthermore, we identify a miR-127-mediated molecular switch that, by modulation of the Bcl6 level and the subsequent assembly of the co-repressor complex, reprogrammed the IRF7 gene from a “locked” to an active state and thus initiated RIG-I-driven signaling. Together, our data establish an unexpected biological role for Bcl6 in coordinating antiviral signaling, which was previously considered to be a major player in lymphocyte development and lymphoma progression^32^,^33^.

Bcl6 is a well-established member of the BTB/POZ zinc finger family with a potential transcriptional regulatory activity. It can directly regulate the transcription of various genes that are essential in cancer, immune and metabolism^34^. Also it can interact with the cofactors and exert its transcriptional regulation on targeted genes^33^. In this sense, the transcriptional program targeted by Bcl6 is suggested to be dependent on the specific co-factors associated in the given context^35^. We, herein, for the first time, demonstrated that Bcl6 interacted with NcoR2 and HDAC3 and assembled to set an activate threshold for antiviral signaling. This association appeared to be functionally cooperative, as specific interference of the interaction between Bcl6 and NcoR2 by RI-BP treatment caused a release of IRF7-driven signaling. In accordance with this, the occupancy of NcoR2 and HDAC3 at the IRF7 loci showed to coincide with that of Bcl6 but correlate inversely with the chromatin accessibility of IRF7 gene (Fig. 3a,d). Notably, although NcoR2 and HDAC3 remained at a high level over the infection period, they failed to reside on IRF7 promoter and hinder its transcription when Bcl6 expression was suppressed by miR-127. The data thus led to a unique regulatory mode whereby the Bcl6 inhibitory complex dynamically controlled the priming of antiviral gene program. Indeed, this kind of regulation has been described in TLR signaling. It has been reported that TLR4-targeted genes were occupied by both stalled RNA pol II and NCoR1 under basal conditions. A signal-dependent removal of the regulatory complex or a lack of NCoR1 was able to de-repress LPS-responsive genes. Also, a CaMKII-initiated clearance of the preexisting repressive complex was proposed to be the prerequisite for TLR2 activation^2^,^36^. Thus, the data, combined with our current finding, may suggest a general requirement for the immune system to transform the pathogenic information into a regulatory code to facilitate the key genes from poised/repressed to actively transcribed states^37^,^38^.

In the search of the molecular bridge that delivered the viral information to the Bcl6 regulation, we identify miR-127, a RIG-I-inducible miRNA, as a strong candidate. MiR-127 has been established as a key player in embryogenesis, oncogenesis and inflammation. Our previous study indicated that it was essential in polarized macrophage development and thus inflammatory disorders. Additionally, miR-127 was found to be induced by viral infection, such as EBV and HBV infection, and was implicated in Burkitt lymphoma pathogenesis^39^. The data suggest a potential role for miR-127 in host-virus interaction. We herein provide evidences to support its importance in promoting RIG-I-induced antiviral signaling, which is presumably achieved by down-regulation of Bcl6 expression and hence the release of IRF7 restraint. Tight control of miR-127 expression is thus essential to restrain antiviral signaling in resting or temporarily activated macrophages. Interestingly, we found that a low level of miR-127 appeared to be “leaked” from the resting macrophages compared with its level at 2 h post infection (Fig. 5a), implying that the signaling driving IFN production is not completely locked in the basal condition. Concurrent with this, the peaked Bcl6 expression did not occur till 2-3 h post infection (Fig. 1a,b). This interesting phenomenon may provide a possible explanation for the well-recognized phenomenon that IFN signaling is inherently activated in the physiological setting, though to a low degree^40^. However, it should be noted that, by elevating the Bcl6 level and forming the inhibitory compound at the key signaling molecule, the host immune system establishes a strict threshold and ensures that the full antiviral response is ‘locked in’ until a full-fledged stimulus occurs^41^. Moreover, our data demonstrated a critical requirement of IFNβ and NF-κB activity in the induction of miR-127 upon viral infection. This may lead to the assumption that the small amount of type I IFN produced in resting or initially activated cells is able to promote miR-127 expression, which in turn enhance antiviral signaling by removing Bcl6-mediated IRF7 repression. This positive feedback loop is believed to be required for optimal antiviral immunity. On the other hand, our data revealed that miR-127 exerted an unexpectedly inhibitory role on NF-κB activation, which, combined with the observed NF-κB-dependent miR-127 generation, constitutes a negative feedback mechanism to control overzealous antiviral gene activation. Thus, miR-127 and Bcl6 exert a fine-tuning effect on antiviral innate immunity and thus have a profound impact on virus-associated diseases (Fig. 5k).

A successful antiviral defense requires the host to restrict virus replication while preventing inflammatory injury. An imbalance between antiviral gene expression and pro-inflammatory response may lead to lethal viral infections^42^. Of interest, our present study revealed an unexpected role for Bcl6 in promoting the inflammatory sequelae of antiviral responses. This is likely counter-intuitive, as Bcl6 has been previously established as a negative regulator of NF-κB-mediated pro-inflammatory signaling upon TLR activation^15^. Although the mechanism responsible for this modulation remains elusive at this stage, one possible explanation for this discrepancy may lie in the different action mode of Bcl6 in the different condition^43^. As proposed by Barish *et al*. Bcl6 and NF-κB occupy at the same proinflammatory genes upon TLR4 engagement, which may cause the counteracting effect between these two factors. However, our data show that, upon RIG-I engagement, Bcl6 primarily acts on IRF7 not on NF-κB-targeting genes, which may prevent the antagonizing effect between them. Moreover, IRF7 itself was recently found to have a negative effect on NF-κB activation, and repressing IRF7 expression accordingly caused the pathological inflammation^44^,^45^. It thus can be postulated that Bcl6-mediated restraint of IRF7 transcription may account for its proinflammatory activity. Interestingly, this contradictory modulation of antiviral and pro-inflammatory signaling has been described in the critical immune factors such as promyelocytic leukemia zinc finger (PLZF), neuregulin receptor degradation protein 1 (Nrdp1) and the Src homology region 2 domain-containing tyrosine phosphatase-1 (SHP-1)^46^,^47^,^48^. More importantly, this proinflammatory action of Bcl6 appeared to be relevant to a well-known clinic entity, diffuse large B-cell lymphoma associated with chronic inflammation (DLBCL-CI), which was characterized by high Bcl6 level and simultaneously sustained inflammation^49^. Also, Bcl6 + Tfh cells was found to be implicated in the pathogeneses of inflammatory disease^50^,^51^. Given the little knowledge about Bcl6-associated inflammatory pathology currently, our finding may provide a novel insight on these conditions.

In summary, our present study identifies that Bcl6, by interacting with the co-factors NcoR2 and HDAC3, plays a pivotal role in controlling IRF7 induction and antiviral signaling priming. Signal-dependent clearance of the Bcl6, largely dependent on the miR-127 induction, led to the optimal activation of antiviral signaling and the alleviated inflammatory response. The data may represent a novel regulatory circuitry during RNA virus infection, and offer a potential molecular target for controlling viral and other related diseases^32^,^52^.

## Methods

### Reagents

The antibodies against Bcl6, HDAC3, β-actin, ERK, p38, JNK, p65 and IκBα were purchased from Santa Cruz Biotechnology (Santa Cruz, CA). The anti-NcoR2 antibody was obtained from Thermo Scientific (Rockford, IL) and the anti-IRF7 antibody was from Invitrogen (Carlsbad, CA). The antibodies to H3K9ac and H3K27ac were obtained from Abcam (Cambridge, MA). The p-ERK (Thr202/Tyr204), p-p38 (Thr180/Tyr182), p-JNK (Thr183/Tyr185), p-IκBα (Ser32), p-p65 (Ser468) and p-IRF3 (Ser396) and anti-RIG-I antibodies were from Cell Signaling Technology (Beverly, MA). Poly I:C was purchased from Sigma-Aldrich (St. Louis, MO). The RI-BPI and the control peptide (CP) were synthesized by Chinese Peptide co. (Hangzhou, China), as described previously^30^. The pGL-3 and pRL-TK-Renilla luciferase plasmids were from Promega (Madison, WI). Unless indicated otherwise, all tissue culture reagents including DMEM culture medium, penicillin/streptomycin were purchased from Invitrogen.

### RNA quantification

Total RNA was isolated using TRIzol reagent (Invitrogen) following the manufacturer’s protocol. 1 μg of total RNA were used for each sample and converted into cDNA using First Strand Kit (Qiagen). SYBR Green PCR Master Mix (Bio-Rad) was used to detect the mRNA levels, and the relative expression levels were determined by applying the ΔΔCt method, using β-actin or U6 as the endogenous control. The primer sequences are provided in the supplementary material.

### Cell culture and peritoneal macrophage isolation

RAW264.7, HEK293T and THP-1 cells were obtained from American Type Culture Collection (Rockville, MD). The cells was grown in Dulbecco’s modified Eagle medium supplemented with 100 U ml^−1^ penicillin, 100 μg ml^−1^ streptomycin, and 10% heat-inactivated fetal bovine serum. To prepare murine peritoneal macrophages, 8-wk-old mice were injected i.p. with 2-3 ml of 3% thioglycolate broth. After 72 h, the peritoneal cells were harvested, and macrophages were enriched by quick adherence.

### Virus infection and viral loads evaluation

RAW264.7 or murine peritoneal cells were infected with VSV (multiplicity of infection (MOI) = 1) for the indicated period of time. Alternatively, RAW264.7 cells were transfected with miR-127, anti-miR-127 or the non-specific control (NC) for 24 h and then infected with VSV for the indicated time periods. For the Bcl6 blocking assay, RAW264.7 cells were subjected to a 12-hour treatment of 5 μM of RI-BPI or control peptide (CP) prior to VSV infection^30^. The virus titers were determined by standard plaque assays or by measurement of VSV RNA level using quantitative PCR assay^8^. The primer sequences for VSV RNA are provided in the supplementary material.

### Expression plasmid constructs and RNA interference

A recombinant vector encoding mouse Bcl6 was constructed by PCR-based amplification and sub-cloning into the pcDNA3.1 eukaryotic expression vector (Invitrogen). For Bcl6 knockdown, the expression vectors (SureSilencing shRNA, Qiagen) with the insertions of Bcl6-specific or the scrambled shRNA duplexes were constructed. The target sequences were synthesized as follows: 5′-CAGCCUCUUAUCCCAUGUA-3′. Transfections were performed using the jetPEI reagent (PolyPlus transfection) according to the manufacturer’s instructions. Stable cell lines were selected in 600 μg ml^−1^ G418 for 3–4 weeks. For Bcl6 knockdown in THP-1 cells, siRNAs were transiently electroporated into lymphoma cell lines using cell line nucleofactor transfection kit (AMAXA) according to the manufacturer’s instruction. The sequence for siRNA was as follows: 5′- CCAUUGUGAGAAGUGUAACCUGCAU-3′^53^.

### Histopathology

The control and experimentally infected mouse lungs were processed for hematoxylin and eosin (H&E) staining30. Histological analysis was performed by a blinded pathologist using a modified scoring system as previously described^54^. All lung fields at ×20 magnification were examined for each sample. Assessment of histological lung injury was performed by grading as follows: 1, normal; 2, focal (<50% lung section) interstitial congestion and inflammatory cell infiltration; 3, diffuse (>50% lung section) interstitial congestion and inflammatory cell infiltration; 4, focal (<50% lung section) consolidation and inflammatory cell infiltration; 5, diffuse (>50% lung section) consolidation and inflammatory cell infiltration. The mean score was used for comparison between groups.

### Reporter plasmid construction and luciferase reporter assays

For the IRF7 reporter plasmids, 1.6 kb DNA sequences were amplified and inserted into the pGL3 vector (Promega). Deletion in each of 5 putative binding sites (S1–S5) at IRF7 promoter was generated using the QuickChange Site-Directed Mutagenesis kit (Stratagene). All of the constructs were confirmed with DNA sequencing. The IFNβ, IRSE–luc, pMIR-Bcl6 and pMIR-Bcl6-mut reporter plasmids were previously describe^23^,^55^. For the luciferase reporter assays, HEK293T or RAW264.7 cells were transfected with the reporter plasmids as indicated, along with pRL-TK renilla luciferase plasmids using Jet-PEI (Polyplus Transfection) for 24 h. The firefly luciferase activity was measured with the Dual-Luciferase Reporter Assay System (Promega) and the data were normalized to the renilla luciferase activity.

### Chromatin immunoprecipitation (ChIP) with quantitative PCR

The cells were crosslinked with 1% formaldehyde for 10 min at 37 °C to cross-link the nuclear proteins to DNA. Subsequently, the cells were harvested by centrifugation at 4 °C for 4 min at 1000 × g, and then lysed in 200 μl SDS lysis buffer (1%SDS, 10 mM EDTA and 50 mM Tris-HCl), sheared with a Diagenode Bioruptor to chromatin fragment sizes of 200–1000 base pairs. Chromatin was immunoprecipitated with Bcl6, NcoR2, HDAC3, H3K9ac, H3K27ac or control IgG antibodies (Santa Cruz Biotechnology)^56^. After complete washing, the immunoprecipitated DNA was quantified with real-time PCR. The IRF7-specific primer sequences are as follows: forward 5′-ATCTTGCGCCAAGACAATTCAGGG-3′ and Reverse 5′-TTGTGGCACTGCTCACCAGTAGAT-3′. The data was normalized to input (input %) or to Ig control (enrichment fold). ΔCt (normalized to the input) = (Ct [ChIP] − (Ct [Input] − Log2 (Input Dilution Factor). ΔCt (normalized to Ig) = (Ct [ChIP] − (Ct [Ig]). The relative expression levels were determined by applying the 2-ΔΔCt method.

### Animal experiments

All of the animal experiments were performed in accordance with the National Institutes of Health Guide for the Care and Use of Laboratory Animals and with the approval and monitoring of the Animal Care and Use Committee of School of Medicine at Hangzhou Normal University. The mice were anesthetized intra-peritoneally with ketamine hydrochloride (100 mg kg^−1^) and xylazine (10 mg kg^−1^), and intratracheally (i.t.) infected with VSV (2 × 10^7^ pfu per mouse) for the indicated time periods. For the Bcl6 blocking experiments, the mice were pretreated with RI-BPI or the control peptide (10 mg kg^−1^, i.t.) for 6 h and then infected with VSV. For miR-127 functional analysis, the mice were instilled with miR-127 mimic, anti-miR-127 or their non-specific oligonucleotide controls (2 mg kg^−1^, i.t.) for 24 h, and then challenged with VSV. The virus titers in lungs were determined as described above. The bronchoalveolar lavage fluid (BALF) was recovered, centrifuged, and supernatant was collected for cytokines analysis. The control and experimentally infected mouse lungs were processed for H&E staining^23^.

### Immunoprecipitation and immunoblotting

For immunoprecipitation, cells were lysed and incubated with the appropriate antibodies (anti-Bcl6 or anti-HDAC3) at 4 °C overnight. Antibody complexes were isolated using protein A/G–agarose beads (Pharmacia Biotech) followed by incubation at 4 °C for 3 h, the beads were washed twice with RIPA buffer, once with high salt buffer (0.5M NaCl), and once with Tris buffer (10 mM Tris, pH7.4), then the immunocomplexes were analyzed with SDS–PAGE and western blotting as described previously^56^. The working concentrations of the indicated antibodies were according to the manufacture’s suggestions.

### Cytokine level determinations

The TNF-α and IL-6 levels were measured in the culture supernatants or BALF with ELISA (R&D Systems). Secreted IFNβ protein was measured using a murine or human IFNβ ELISA Kit (PBL Biomedical).

### Statistical analysis

All of the data, unless otherwise indicated, are presented as the means ± SEM of independent experiments. The statistical significance of the differences was analyzed with Student’s *t* test or two-way ANOVA test. A *P* value of 0.05 or less was considered to be statistically significant. All of the calculations were performed using the Prism software program for Windows (GraphPad Software).

## Additional Information

**How to cite this article**: Xu, F. *et al.* Bcl6 Sets a Threshold for Antiviral Signaling by Restraining IRF7 Transcriptional Program. *Sci. Rep.* **6**, 18778; doi: 10.1038/srep18778 (2016).

## Supplementary Material

Supplementary Information
