# Corrigendum: Bcl6 Sets a Threshold for Antiviral Signaling by Restraining IRF7 Transcriptional Program

**DOI:** 10.1038/srep46904

**Published:** 2017-09-20

**Authors:** Feng Xu, Yanhua Kang, Ningtong Zhuang, Zhe Lu, Hang Zhang, Dakang Xu, Yina Ding, Hongping Yin, Liyun Shi

Scientific Reports 6: Article number: 18778; 10.1038/srep18778 published online: 01 05 2016; updated: 09 20 2017.

During the preparation of the figures for this Article, some of the panels were misassembled in the main figures and in the Supplementary Information. The corrected figures are published below, and the corrected Supplementary Information file is appended.

The following changes are reflected in the corrected versions of the figures:

- In Figure 1b, actin bands are replaced. Figure 1c and 1h are replaced completely.

- In Figure 2b, IRF3 bands for pcD and pcBcl6 are replaced. Figure 2c is replaced completely.

- Figure 3b is replaced completely.

- In Figure 4d, IkBα bands for pcD and pcBcl6 are replaced. In Figure 4e actin bands are replaced.

- In Figure 5d, Bcl6 bands are replaced.

- In Figure 6c, Bcl6 and both actin controls are replaced.

Additionally, the following changes are reflected in the corrected version of the SI:

- The original IRF3 blot for shCtl and shBcl6 in Figure 2b is replaced.

- The original actin control blot for Figure 4e is now added.

- Original blots for Figure 5 are now added.

- The original blots for Bcl6 and corresponding actin control for NC and miR-127 in Figure 6h are replaced.

The conclusions of the Article are unaffected by these changes. The authors apologise for the errors and any confusion caused.

## Supplementary Material

Supplementary Information

## Figures and Tables

**Figure 1:**
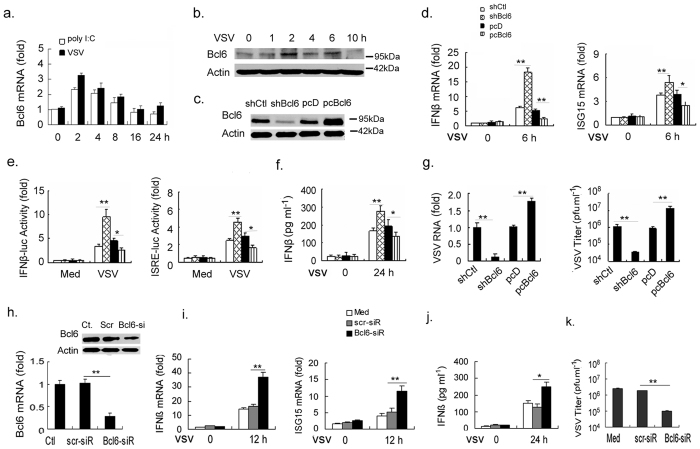
Bcl6 negatively regulates type I IFN production upon RNA virus infection.

**Figure 2:**
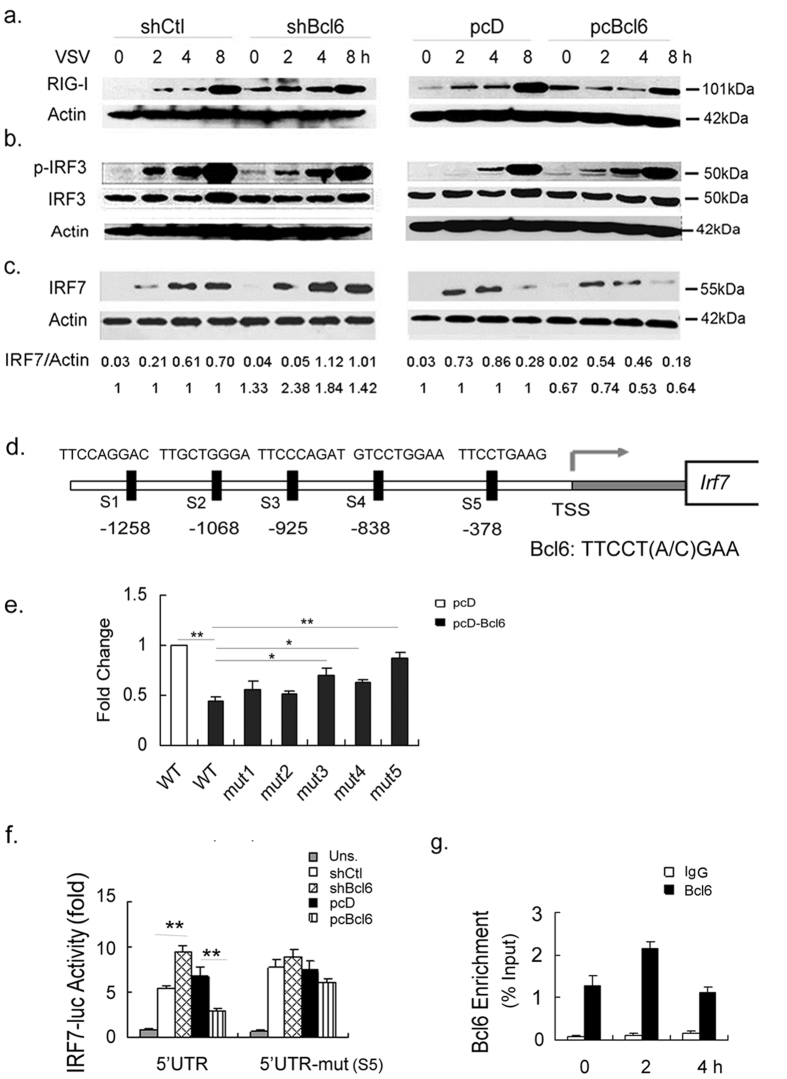
Bcl6 specifically modulates IRF7-driven antiviral signaling.

**Figure 3:**
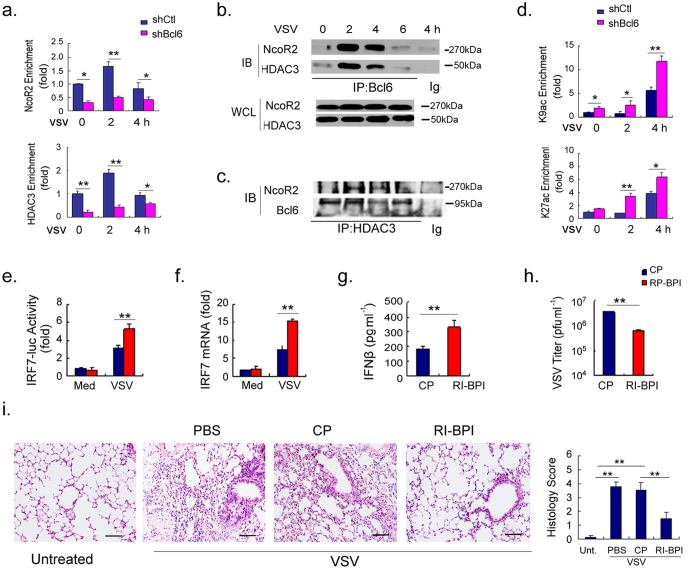
Bcl6 controls IRF7 gene transcription via interaction with HDAC3 and NcoR2.

**Figure 4:**
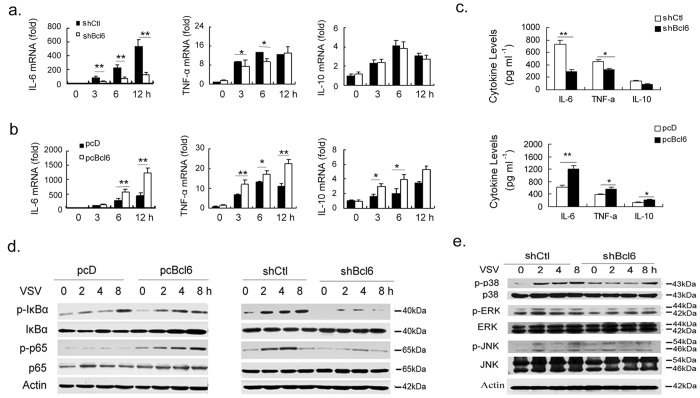
Enhancement of the inflammatory sequelae of antiviral responses by Bcl6.

**Figure 5:**
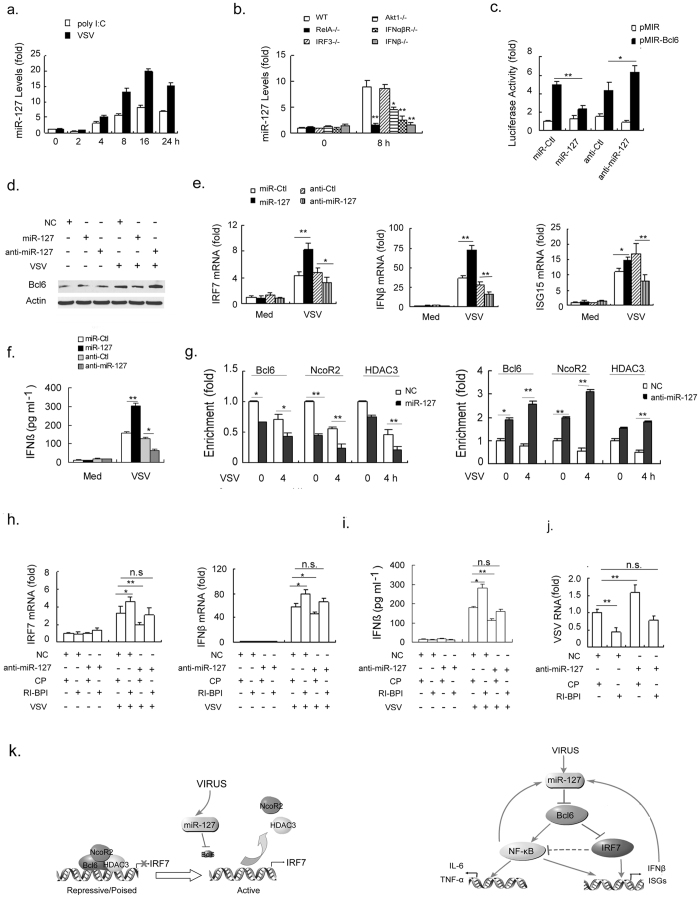
miR-127 mediates the signal-dependent turnover of Bcl6 coregulator.

**Figure 6:**
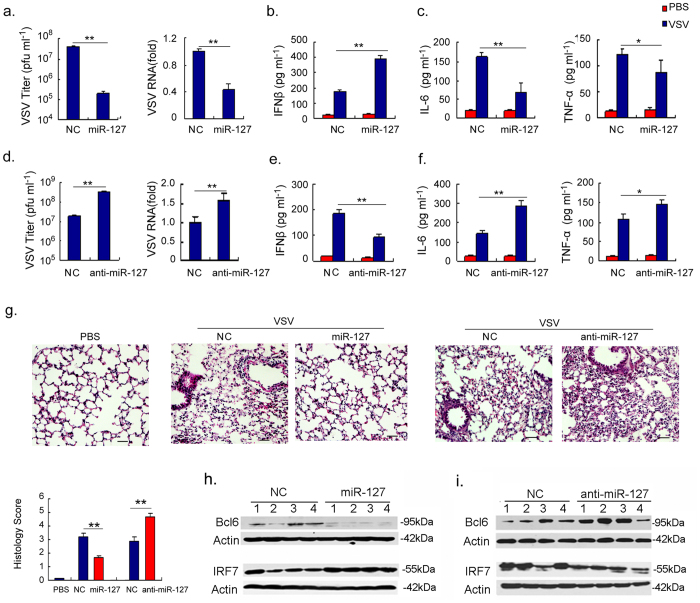
The miR-127-Bcl6-IRF7 circuit regulates the viral immunopathologic response in vivo.

