# Supplementary material for: Bcl6 Sets a Threshold for Antiviral Signaling by Restraining IRF7 Transcriptional Program
Source: Sci Rep. 2016 Jan 5;6:18778. doi: 10.1038/srep18778 (PMC4700451; doi:10.1038/srep18778)

Bcl6 Sets a Threshold for Antiviral Signaling by Restraining IRF7 Transcriptional Program

Feng Xu[1,*], Yanhua Kang[2,*], Ningtong Zhuang[2], Zhe Lu[2], Hang Zhang[2], Dakang Xu[2,3], Yina Ding[2], Hongping Yin[2], Liyun Shi [2,4,#]

1. Department of Infectious Diseases, Second Affiliated Hospital, Zhejiang University School of Medicine, Hangzhou, Zhejiang 310009, China

2. Department of Microbiology and Immunology, Key Lab of Immunology and Molecular Medicine, School of Medicine, Hangzhou Normal University, Hangzhou, Zhejiang 310036, China

3. MIMR-PHI Institute of Medical Research, Clayton, Victoria 3168, Australia

4. Department of Microbiology and Immunology, Nanjing University of Chinese Medicine, Nanjing 210046, China

* These authors contributed equally to this work

[#] Correspondence: Liyun Shi (shi_liyun@msn.com)

Supplementary Figures

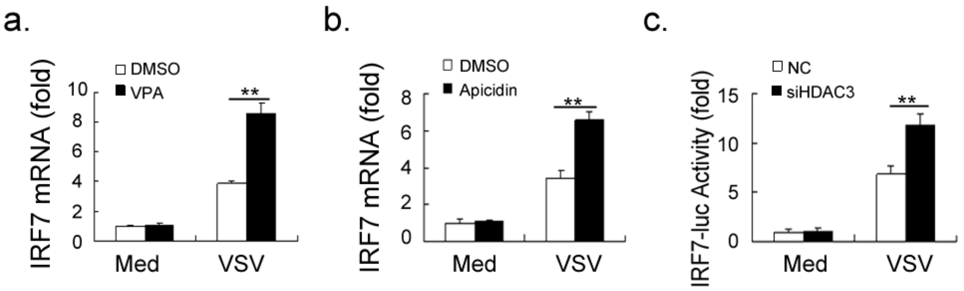

Supplementary Figure 1. The effect of histone deacetylase on IRF7 transcription. (a,b) RAW264.7 cells were pretreated with Valproic acid (VPA, 5mM) or Apicidin (2.5 µM) for 18 h, followed by VSV infection (MOI 1) for 6 h. mRNA level of IRF7 was analyzed by quantitative PCR. **(c)** RAW264.7 cells were transfected with HDAC-siRNA or the non-specific control (NC) for 24 h, and infected with VSV (MOI 1). The activity of IRF7 reporter plasmid was tested by dual-luciferase assay 6 h post infection. Data are representative of three experiments and depicted as means ± SEM. **p < 0.01, *p < 0.05 by student's *t* test.

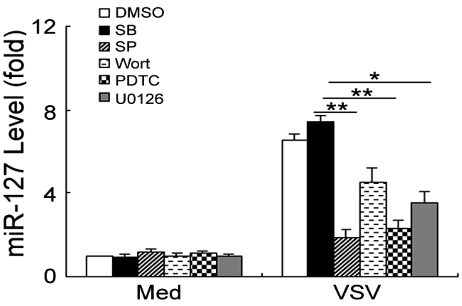

Supplementary Figure 2. The role of NF-κB and MAPKs in miR-127 induction upon VSV infection. RAW264.7 cells were pretreated respectively with the specific inhibitor for NF-κB (PDTC, 10 µM), PI3-K (Wortmanin, 100nM), ERK (UO126,10 µM), JNK (SP600125, 10 µM), p38 (SB239063, 20 µM), or DMSO as control for 30 min, and then infected with VSV for 8h. miR-127 level was measured by quantitative PCR and normalized to that of U6 in each sample. Data are representative of three experiments and depicted as means ± SEM. **p < 0.01, *p < 0.05 by student's *t* test.

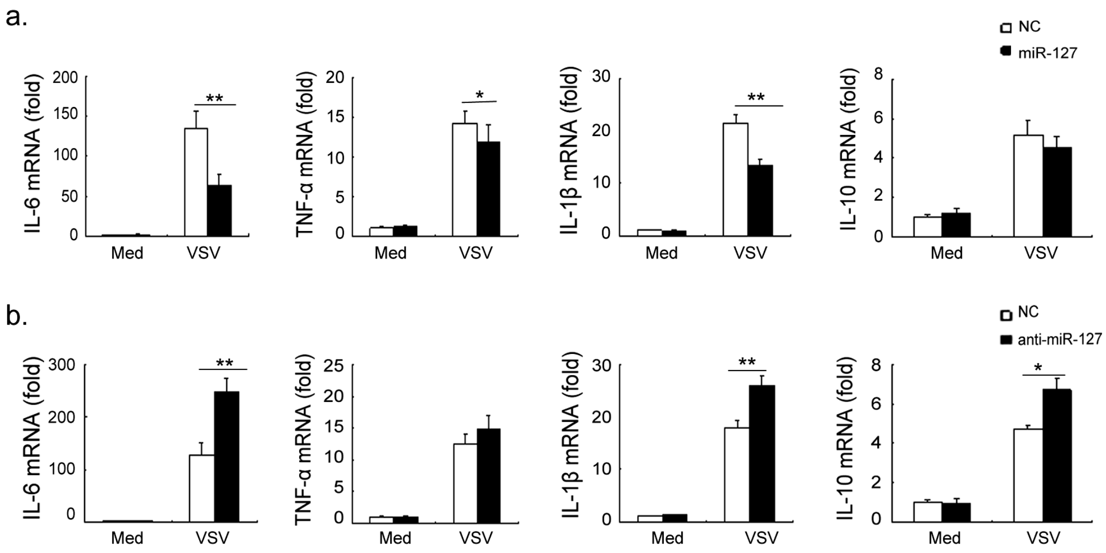

Supplementary Figure 3. The effect of miR-127 on the expression of proinflammatory cytokines triggered by RIG-I engagement. (a,b) RAW264.7 cells were transfected with miR-127, anti-miR-127 or their controls for 24 h, and then infected with VSV (MOI 1) for 6 h. mRNA levels of proinflammatory cytokines were analyzed by quantitative PCR. Data are representative of three experiments and depicted as means ± SEM. $^{**}p < 0.01$, $^{*}p < 0.05$ by student's *t* test.

Supplementary Table 1.

Primer sequences for the quantitative PCR

Gene	Forward(5`-3`)	Reverse(5`-3`)
mTNF-α	AAGGCCGGGGTGTCCTGGAG	AGGCCAGGTGG GGACAGCTC
mIl-6	CCACTTCACAAGTCGGAGGCT	AGTGCATCATCGTTGTTCATAC
mIl-10	CAGAGAAGCATGGCCCAGA	TGCTCCACTGCCTTGCTCTTA
mIFN-β	ATGAGTGGTGGTTGCAGGC	TGACCTTTCAAATGCAGTAGATTCA
mISG15	CAGGACGGTCTTACCCTTTCC	AGGCTCGCTGCAGTTCTGTAC
mIRF7	ACAGGGCGTTTTATCTTGCG	TCCAAGCTCCCGGCTAAGT
mBcl6	CCGGCTCAATAATCTCGTGAA	GGTGCATGTAGAGTGGTGAGTGA
hIFNβ	CATTACCTGAAGGCCAAGGA	CAATTGTCCAGTCCCAGAGG
hISG15	TCCTGGTGAGGAATAACAAGGG	GTCAGCCAGAACAGGTCGTC
VSV	ACGGCGTACTTCCAGATGG	CTCGGTTCAAGATCCAGGT
mActin	CTCATGAAGATCCTGACCGAG	AGTCTAGAGCAACATAGCACAG
miR-127	GCGGCTCGGATCCGTCTGAGCT	GTGCAGGGTCCGAGGT

Uncropped gel images in this study

Figure 1b

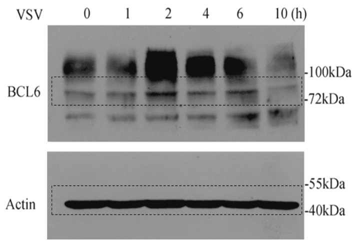

Figure 1c

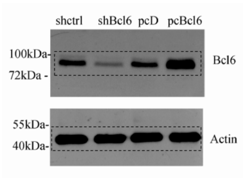

Figure 1h

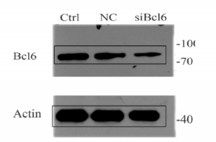

Figure 2a

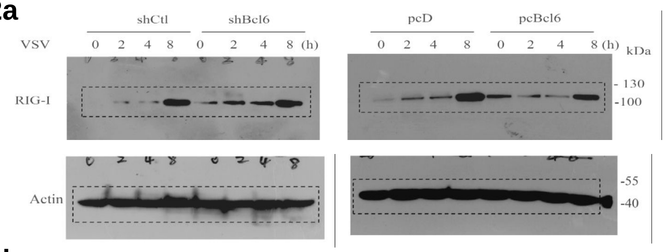

Figure 2b

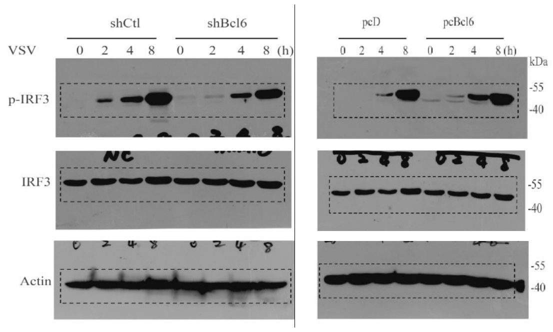

Figure 2c

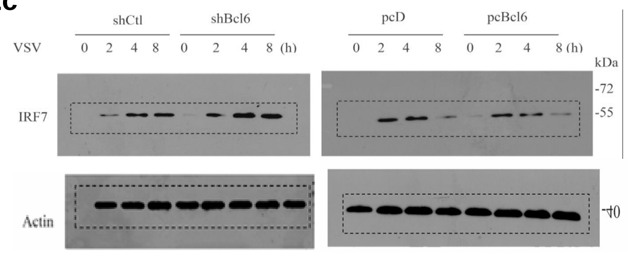

Figure 3b

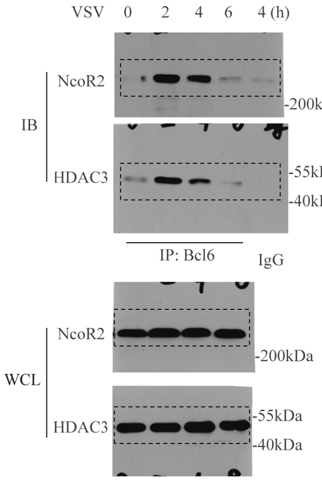

Figure 3c

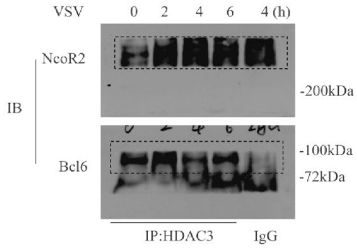

Figure 4d

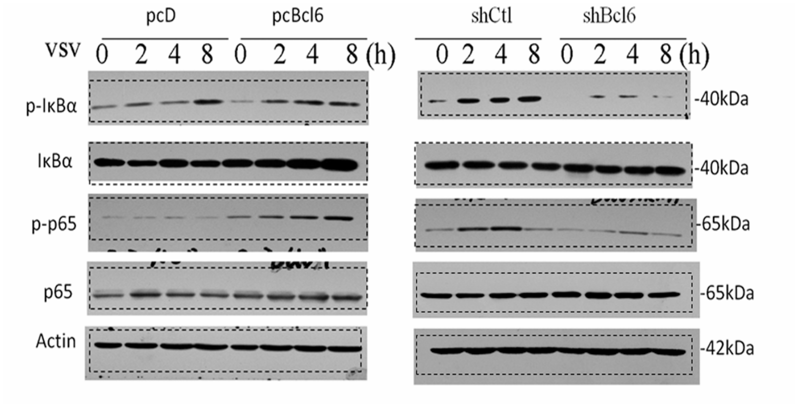

Figure 4e

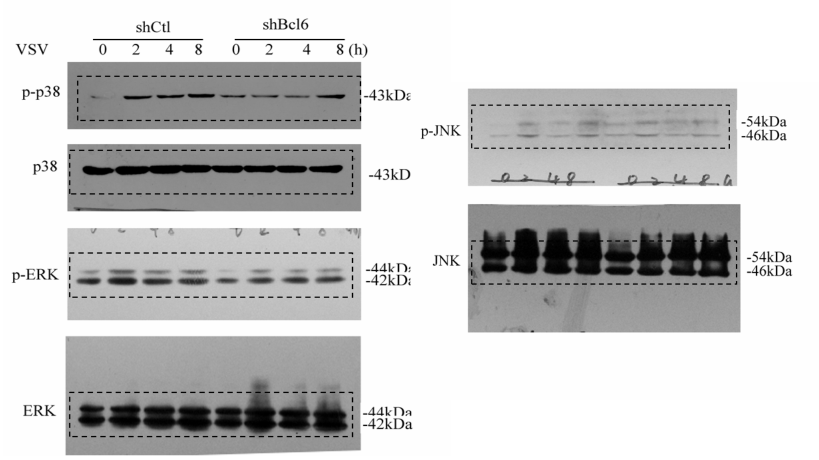

Figure 6h

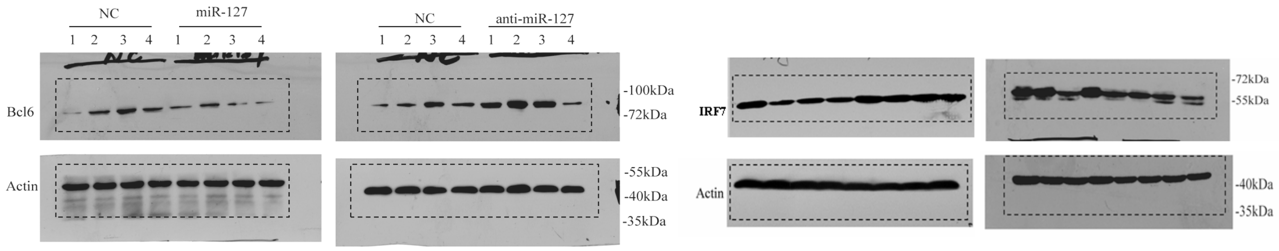

Supplement: Supplementary Information [file srep18778-s1.pdf]
